# Promoting Immortalized Adipose-Derived Stem Cell Transdifferentiation and Proliferation into Neuronal-Like Cells through Consecutive 525 nm and 825 nm Photobiomodulation

**DOI:** 10.1155/2022/2744789

**Published:** 2022-09-05

**Authors:** Madeleen Jansen van Rensburg, Anine Crous, Heidi Abrahamse

**Affiliations:** Laser Research Centre, Faculty of Health Sciences, University of Johannesburg, P.O. Box 17011, Doornfontein, Johannesburg, South Africa 2028

## Abstract

Neuronal cells can be generated from adipose-derived stem cells (ADSCs) through biological or chemical inducers. Research has shown that this process may be optimized by the introduction of laser irradiation in the form of photobiomodulation (PBM) to cells. This *in vitro* study is aimed at generating neuronal-like cells with inducers, chemical or biological, and at furthermore treating these transdifferentiating cells with consecutive PBM of a 525 nm green (G) laser and 825 nm near-infrared (NIR) laser light with a fluence of 10 J/cm^2^. Cells were exposed to induction type 1 (IT1): 3-isobutyl-1-methylxanthine (IBMX) (0.5 mM)+indomethacin (200 *μ*M)+insulin (5 *μ*g/ml) for 14 days, preinduced with *β*-mercaptoethanol (BME) (1 mM) for two days, and then incubated with IT2: *β*-hydroxyanisole (BHA) (100 *μ*M)+retinoic acid (RA) (10-6 M)+epidermal growth factor (EGF) (10 ng/ml)+basic fibroblast growth factor (bFGF) (10 ng/ml) for 14 days and preinduced with *β*-mercaptoethanol (BME) (1 mM) for two days and then incubated with indomethacin (200 *μ*M)+RA (1 *μ*M)+forskolin (10 *μ*M) for 14 days. The results were evaluated through morphological observations, viability, proliferation, and migration studies, 24 h, 48 h, and 7 days post-PBM. The protein detection of an early neuronal marker, neuron-specific enolase (NSE), and late, ciliary neurotrophic factor (CNTF), was determined with enzyme-linked immunosorbent assays (ELISAs). The genetic expression was also explored through real-time PCR. Results indicated differentiation in all experimental groups; however, cells that were preinduced showed higher proliferation and a higher differentiation rate than the group that was not preinduced. Within the preinduced groups, results indicated that cells treated with IT2 and consecutive PBM upregulated differentiation the most morphologically and physiologically.

## 1. Introduction

Within the field of regenerative medicine, stem cell (SC) technology has increasingly become a hot topic [1]. This is due to the self-renewal properties, expansion capabilities, and differentiation possibilities of SCs. Of these cells, adipose-derived stem cells (ADSCs) are becoming the cell type of choice due to their abundant availability and the ease at which they can be sourced through surgery that is not as invasive to donors as other cell types may require [2]. Furthermore, preliminary research performed *in vivo* and *in vitro* has shown that ADSCs can differentiate into multiple phenotypes, such as osteoblasts, or chondrocytes, depending on the inducers applied [3]. Specifically, ADSCs can transdifferentiate into neuron-like cells by applying a wide range of inducers that include forskolin, insulin, brain-derived growth factor (BDNF), or even retinoic acid (RA) [4]. ADSCs show potential for clinical use in the future to aid in the repair of mechanical brain injuries and neurodegenerative diseases [2, 5, 6]. This study used immortalized ADSCs (iADSCs) as previous studies have established iADSCs as an alternative model to isolated ADSCs while yielding similar results [7].

The metabolism of cells can be stimulated or inhibited by laser irradiation in the form of photobiomodulation (PBM) [8]. Whether the irradiation treatment is stimulatory or inhibitory is dependent on the laser parameters that are used. Wavelengths that range from visible to near-infrared (NIR) light (400-1100 nm) and the fluence (J/cm^2^) are included in these parameters [2, 9–12]. It should be noted that 3–5 J/cm^2^ is the most effective fluence for stimulating proliferation, whereas fluences higher than this can prove to be overstimulatory and fluences lower than this is ineffective [1, 2]. Photobiomodulation has a stimulatory effect on mitochondrial chromophores, which in turn increases the activity of the electron transport chain and leads to an overall increase in adenosine triphosphate (ATP) production which upregulates the mitochondrial membrane potential (MMP) [13]. Various studies have shown that visible spectrum light, 450 nm–580 nm, stimulates an increase in differentiation from ADSCs into osteoblasts [1, 14, 15]. Furthermore, green laser irradiation has been shown to aid in the triggering of differentiation transcription factors [16]. While blue and green laser irradiation does not significantly stimulate proliferation, it has been shown in numerous studies that red and NIR laser light, such as 660 nm and 810 nm, upregulates proliferation significantly in ADSCs [8, 9, 17, 18]. Near-infrared laser irradiation increases the homing abilities of ADSCs to injured sites by significantly increasing the migration of ADSCs [18, 19]. The current study observed the effect on iADSC transdifferentiation into neuronal-like cells when a novel combination of consecutive laser light of 825 nm and 525 nm at 10 J/cm^2^ was used. The effect of these irradiation parameters on morphology, migratory abilities, cellular viability, proliferation, and genetic expression was observed.

## 2. Materials and Methods

### 2.1. Cell Culture

Adipose-derived stem cells immortalized with hTERT ASC52Telo (ATCC® SCRC-4000™) were cultivated in Dulbecco's Modified Eagle Media (DMEM) (Sigma-Aldrich, D5796) fortified with 0.5% amphotericin B solution (Sigma-Aldrich, A2942), 0.5% penicillin-streptomycin (Sigma-Aldrich, P4333), and 10% foetal bovine serum (FBS Superior) (Biochrom, S0615). Maintenance of cells was upheld in 85% humidity at 37°C and 5% CO_2_ (Heracell™ 150i CO_2_ Incubator, Thermo Scientific™, 51026280) in Corning® cell culture flasks (Sigma, CLS430639/CLS430641/CLS431080).

### 2.2. Photobiomodulation

Once confluent, cultured iADSCs were seeded at a density of 1 × 10^5^ onto 35 mm diameter culture dishes (Corning, 430165) in complete medium and maintained for 24 h to allow adhesion prior to irradiation. After this incubation period, cells were exposed to an 825 nm NIR Diode Laser (National Laser Centre of South Africa, SN 070900108) with a 1000 mA LaserSource (Arroyo Instruments, 4210) and subsequently to a 525 nm green (G) Diode Laser (National Laser Centre of South Africa, EN 60825-1 : 2007) with a 100-240 VAC, 47-63 Hz 5A LaserSource (OptoElectronics Tech.CO., LTD). The power of the laser output (mW) was measured using a FieldMate Laser Power Meter (Coherent, 1098297). Finally, a High-Sensitivity Thermopile Sensor PM3 (Coherent, 1098336) was used to determine the laser irradiation time based on fluency. The laser parameters can be seen in Table 1.

Two main study groups were used concerning PBM: (1) a control group that was not treated with laser light and (2) consecutive treatment with 825 nm and 525 nm (NIR-G) at 5 J/cm^2^ each. The irradiation time of each laser wavelength was calculated with the following formula seen in the following equation:

(1) $$\begin{array}{c} \frac{\text{mW}}{\text{ c}{\text{m}}^{2}}=\frac{\text{mW}}{\pi r^{2}},\\ \frac{\text{W}}{{\text{cm}}^{2}}=\frac{\text{mW}/\text{c}{\text{m}}^{2}}{1000},\\ \text{Time }\left(\text{seconds}\right)=\frac{\text{J}/{\text{cm}}^{2}}{\text{W}/{\text{cm}}^{2}}. \end{array}$$

### 2.3. Characterization of Markers

#### 2.3.1. Flow Cytometry

The CD and neuronal markers of transdifferentiated iADSCs were characterized with flow cytometry (BD Accuri Flow Cytometer, BD Biosciences) through secondary antibody conjugation to a fluorescent marker. According to the ATCC® guidelines, CD44 (Sigma, SAB1405590), CD90 (Sigma, SAB4200497), and CD166 (Sigma, SAB1306488) were screened. Immortalized ADSCs were also screened for early, NSE (Sigma, SAB4200571) and NeuN (Sigma, MAB377), and late, MAP2 (Sigma, M9942) and Tau (Sigma, T9450) neuronal markers, 7 days post-PBM. Cells were treated in suspension at a seeding density of1 × 10^5^and initially washed by centrifugation with ice-cold washing buffer (azide/PBS/BSA: 0.01%*w*/*v*sodium azide (Sigma-Aldrich, S8032)), phosphate buffer solution (PBS), and 0.1%*w*/*v*Bovine Serum Albumin (BSA) (Sigma-Aldrich, A2153). The suspended cells were treated with blocking buffer (BSA/PBS: 10% *w*/*v* BSA, PBS) and incubated on ice for 30 min. The washing step described above was repeated three times. The cells were treated with primary mouse or rabbit anti human antibody in working buffer (azide/PBS/BSA/FBS: 0.01%*w*/*v*sodium azide (Sigma-Aldrich, S8032)), PBS, 0.1%*w*/*v*BSA, and 2% FBS (Biochrom, S0615) accordingly on ice for 30 min. The washing step was repeated three times. The cells were then incubated 30 min on ice in the dark with secondary fluorescent antibodies: fluorescein isothiocyanate (FITC) goat anti-rabbit (NovusBio, NB720-F) for the CD 166 sample and FITC Goat anti-Mouse (NB720-F, NovusBio) for the rest of the samples. The washing step was again repeated. Antigenically, cells were detected through flow cytometry (BD Accuri Flow Cytometer, BD Biosciences) by the conjugated fluorescent markers to determine the presence or absence of the CD and neuronal markers.

### 2.4. Morphology

#### 2.4.1. Inverted Light Microscopy

Morphological changes were observed and analysed 24 h, 48 h, and 7 days following laser exposure with an inverted light microscope (OLYMPUS, CKX41); images were taken with a digital camera (OLYMPUS, SC30) connected to the microscope coupled with CellSens software.

#### 2.4.2. Cell Migration

The “central scratch” method was used to determine the migration of the cells. Immortalized ADSCs were incubated overnight at 37°C, 5% CO_2_, and 85% humidity in 35 mm petri dishes. A scratch was made through the centre of each plate with a sterile P-200 pipette tip, after which the media were changed, and the cells were irradiated. Cellular motility was analysed with an Inverted Microscope (Wirsam, Olympus CKX41) on predetermined positions and focal planes and finally imaged with a digital camera (SC30 Olympus Camera) at 0 h, 24 h, 48 h, and 7 days after laser exposure.

### 2.5. Biochemical Analysis

#### 2.5.1. Viability: MTT Assay

Cellular viability can be measured with a 3-(4,5-dimethylthiazol-2-yl)-2,5-diphenyl tetrazolium bromide- (MTT-) based assay that specifically targets mitochondrial dehydrogenase. Cells, seeded at 1 × 10^5^ seeding density, were treated with a 1 : 10 dilution of reconstituted MTT reagent (5 mg/ml) (TOX1-1KT, Merck/Sigma) and incubated for 4 h at 5% CO_2_, 37°C, and 85% humidity. Complete media were used for the control group. A 1 : 1 ratio MTT solubilization solution (or DMSO) was used to dissolve formazan crystal formation following the incubation period. Finally, a luminometer (Perkin Elmer, Victor3) was used to determine the Relative Light Units (RLU) at 570 nm.

#### 2.5.2. DNA Synthesis

Thymidine is interchangeable with 5-ethynyl-2′-deoxyuridine (EdU) and can thus be incorporated into DNA instead of thymidine in the replication process. This can then be used to measure cellular proliferation by detecting how much EdU was inserted into the DNA. Cells were cultured at a cell seeding density of 1 × 10^5^ and incubated for 24 h with fluorescent azide. Following incubation, the iADSCs were labelled with immunofluorescent markers. A 10% solution of paraformaldehyde (P6148, Sigma-Aldrich) was used to fix cells for 10 min. A 2% BSA solution was used to permeabilize cells for 30 min at room temperature (RT); cells were subsequently washed with PBS three times. Cells were then treated with (1 : 200) *α* actin primary antibody and incubated at RT for 30 min. The treated iADSCs were rinsed three times with ice-cold washing buffer (azide/PBS/BSA: 0.01% *w*/*v* sodium azide (Sigma-Aldrich, S8032)), PBS, and 0.1% *w*/*v* BSA (Sigma-Aldrich, A2153) three times. Then, cells were incubated with donkey anti-mouse Alexa fluor 594 secondary antibodies (1 : 800) at RT for 30 min. Cells were again treated with washing buffer and counterstained with 300 nm 4′,6-diamidino-2-phenylindole (DAPI). The counterstained cells were mounted with Fluoromount™ onto microscopic glass cover slides and observed with an Axio Observer Z1 (Carl Zeiss) microscope.

### 2.6. Protein Detection

#### 2.6.1. ELISA

The early and late neuronal markers, NSE and CNTF, were detected with the NSE *in vitro* SimpleStep ELISA® (Abcam, ab217778) and CNTF *in vitro* SimpleStep ELISA® (Abcam, ab264608) kits, respectively. These kits allow for the quantification of NSE and CNTF proteins within samples. The sample size was *n* = 3, with a duplicate for each sample. Total protein concentration was determined with the Bradford Assay. The SimpleStep kit makes use of an affinity tagged capture antibody and a conjugated reporter detector antibody. The complex is coated to the well through an immunoaffinity anti-tag antibody. The signal generated is proportional to the amount of bound analyte. The absorbance can be measured at 450 nm.

### 2.7. Genetic Expression

#### 2.7.1. RT-PCR

The Human Neurogenesis RT^2^ Profiler PCR Array 96-Well Format (QIAGEN, 330231 PAHS-404ZA) was used to determine the level of expression of 84 genes related to neurogenesis. Immortalized ADSCs were harvested, and RNA extraction was performed using the RNeasy® Mini Kit (QIAGEN, 74104). Then, cDNA was synthesized from the extracted RNA using the RT^2^ First Strand Kit (QIAGEN, 330401). Finally, the RT-PCR experiment was performed with RT^2^ SYBR Green Mastermix (QIAGEN, 330504) with an Agilent Aria MX Real-Time PCR (G8830-64001, Agilent Technologies). This experiment was performed in conjunction with the MIQE guidelines. Results were analysed using the free online QIAGEN GeneGlobe analysis software. The GeneGlobe software calculated the fold change and *P* values using the *ΔΔ*CT method, where *Δ*CT is calculated between the genes and the average of the reference genes. The *ΔΔ*CT is determined through subtraction of the experimental group from the control group, and the fold change can be determined with the 2^(-*ΔΔ*CT) formula.

### 2.8. Statistical Analysis

All laboratory tests (*n* = 4) were repeated four times. Biochemical assays were carried out twice, with the average of the results used. To guarantee the results' validity, all controls were included. The mean, standard deviation, standard error, and significant changes were calculated using SigmaPlot software version 12 for statistical analysis. The statistical difference between the standard and experimental groups was determined using a Student *t*-test. Within a specific period, the ANOVA repeated measure was used to determine statistical significance between the control and experimental groups, as well as the standard and all IT groups, and to compare the groups (controls with controls, etc.). Tables and graphs show statistical differences between the untreated controls and experimental groups as *P*0.05 (∗), *P*0.01 (∗∗), and *P*0.001 (∗∗∗), with dispersion bars representing standard error. Purple stars (∗) indicated experimental samples compared to the control; black stars (∗) indicated samples compared to the standard.

## 3. Results

### 3.1. Characterization of Markers

**Figure 1:**
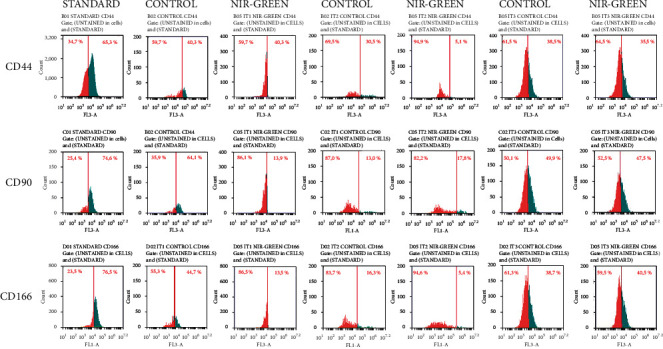
Characterization of differentiated iADSCs using CD44, CD90, and CD166 markers. Fluorescent protein detection through flow cytometry. Flow cytometry analysis revealed a decrease in SC markers 7 days postlaser treatment in experimental groups. A shift to the right in distribution indicated an increase in CD marker expression.

**Figure 2:**
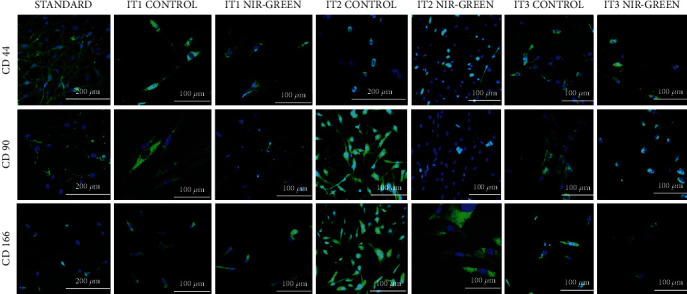
Characterization of differentiated iADSCs using CD44, CD90, and CD166 markers. Fluorescent protein detection through fluorescent microscopy. Fluorescent microscopy revealed the presence of SC markers 7 days post-PBM. Scale bar: 100 *μ*m.

**Figure 3:**
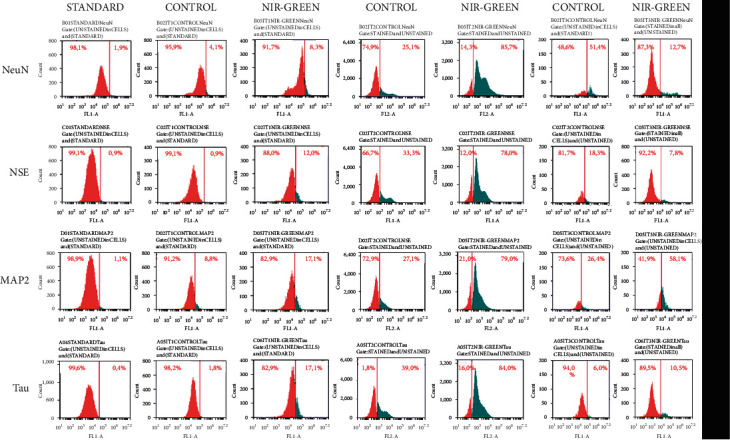
Characterization of transdifferentiated iADSCs using neuronal markers NeuN, NSE, MAP2, and Tau. Fluorescent protein detection through flow cytometry. Flow cytometry analysis revealed an increase in neuronal markers 7 days postlaser treatment. A shift to the right in distribution indicated an increase in neuronal marker expression.

**Figure 4:**
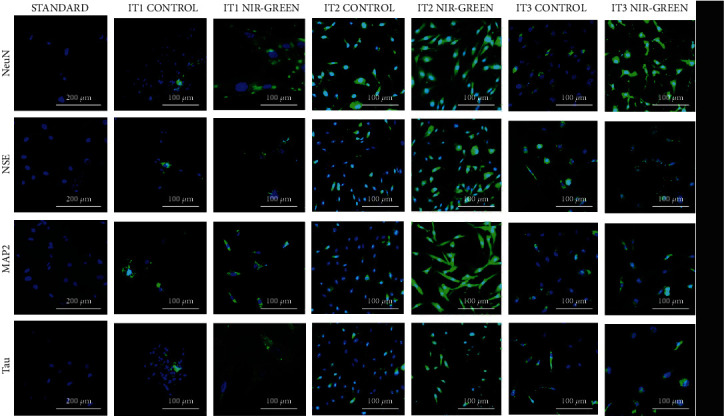
Characterization of early, NSE and NeuN, neuronal markers following transdifferentiation media and consecutive laser irradiation treatment. Fluorescent protein detection through fluorescent microscopy. Fluorescent microscopy revealed the presence of neuronal markers 7 days post-PBM. Scale bar: 100 *μ*m.

Flow cytometry was used to characterize the IT1, IT2, and IT3 NIR-G PBM-treated cells to establish whether iADSCs preserved or lost stemness towards neuronal-like cells and at what percentage this occurred. Three iADSC CD markers (CD44, CD90, and CD166) and two early (NeuN and NSE) and two late (MAP2 and Tau) neuronal markers were applied. The selected CD markers are widely recognized as markers for iADSC SC maintenance. In Figure 1, the standard group indicated high percentages of CD44, CD90, and CD166 at 65.3%, 74.6%, and 76.5%, respectively. A steady decline in SC expression was observed in the control groups. This decline was even more distinct in the experimental groups. Immortalized ADSCs treated with IT1 media and NIR-G PBM showed a significant (*P* < 0.05) decrease in CD44 (40.3%), CD90 (13.9%), and CD166 (13.5%) compared to the IT1 control which maintained CD44 at 40.3%, CD90 at 64.1%, and CD166 44.7%. Immortalized ADSCs treated with IT2 media and NIR-G PBM showed a significant (*P* < 0.05) decrease in CD44 (5.1%), CD90 (17.8%), and CD166 (16.3%) compared to the IT2 control group with CD44 (30.5%), CD90 (13%), and CD166 (5.4%), respectively. The IT3 NIR-G group also demonstrated a decrease in CD markers: CD44 (35.5%), CD90 (47.5%), and CD166 (40.5%) compared to its respective IT3 control group at CD44 (38.5%), CD90 (49.9%), and CD166 (38.7%). Cells treated with differentiation media and laser light indicated immunofluorescence for CD44, CD90, and CD166 SC markers (Figure 2). The SC marker immunofluorescence results did not necessarily signify a lack of differentiation but could also have showed subpopulations of iADSCs. The expression of early and late neuronal markers was detected with flow cytometry to measure the efficacy of transdifferentiation through transdifferentiation media and PBM. In Figure 3, a small percentage of NeuN and NSE were detected in the standard sample (1.9% and 0.9%, respectively). The IT1 control group showed small increments of NeuN and NSE expression (4.1% and 0.9%). After IT1 NIR-G PBM treatment, large increases in NeuN and NSE were noted (8.3% and 12.0%, respectively). A small percentage of MAP2 and Tau were detected in the standard sample (1.1% and 0.4%, respectively). The IT1 control group showed a small upregulation in MAP2 and Tau expression (8.8% and 1.8%, respectively). After IT1 PBM treatment, large increases in MAP2 (17.1%) and Tau (17.1%) expression were observed compared to the standard and control groups. Immortalized ADSCs treated with NIR-G PBM indicated a significant (*P* < 0.05) increase in MAP2 expression with an increase to 17.1% in comparison to the standard (1.1%). The IT2 control group showed an increase in NeuN and NSE expression (25.1% and 33.3%). Immortalized ADSCs treated with IT2 NIR-G PBM that resulted in large increases in NeuN (85.7%) and NSE (78.0%) were observed. The IT2 control group showed small increases in MAP2 and Tau expression (27.1% and 39.0%, respectively). Immortalized ADSCs treated with IT2 NIR-G PBM resulted in large upregulation of MAP2 (79.0%) and Tau (84.0%) compared to the standard and control groups. The IT3 control group showed small increases in MAP2 and Tau expression (26.4% and 6.0%, respectively). Immortalized ADSCs treated with IT3 NIR-G PBM indicated large upregulation of MAP2 (58.1%) and Tau (10.5%) compared to the standard and control groups. Cells treated with transdifferentiation media and PBM demonstrated immunofluorescent expression of neuronal markers NeuN, NSE, MAP2, and Tau (Figure 4).

### 3.2. Morphology

#### 3.2.1. Inverted Light Microscopy

**Figure 5:**
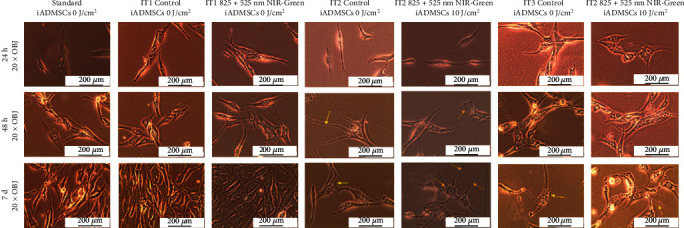
Morphology of transdifferentiated iADSCs 24 h, 48 h, and 7 days postlaser irradiation. Shown here are the untreated standard iADSCs; untreated IT1 control cells, experimental IT1 iADSCs exposed to NIR-G PBM consecutively, untreated IT2 control cells, experimental IT2 iADSCs exposed to NIR-G PBM consecutively, untreated IT3 control cells, and experimental IT3 iADSCs exposed to NIR-G PBM consecutively. Scale bar: 100 *μ*m.

Morphological studies revealed that the standard group had the typical shape observed in healthy iADSCs with a smooth surface and spindle-like shape (Figure 5). After 24 h, treated cells showed cytoplasmic extension. Following 48 h, the treated cells developed distinct changes in morphology; the formation of branches and refraction could be observed. After 7 days, cells transdifferentiated with NIR-G PBM showed morphology changes that were completely different to the standard untreated iADSCs.

#### 3.2.2. Migration

**Figure 6:**
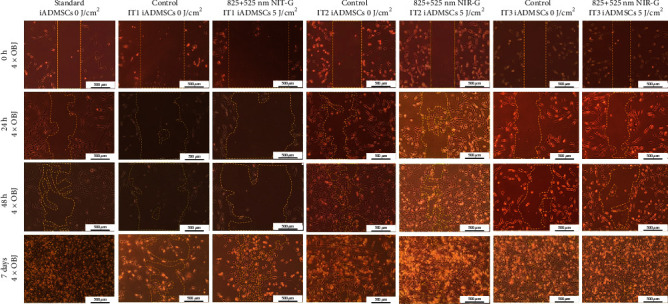
Migration of transdifferentiated iADSCs 0 h, 24 h, 48 h, and 7 days post-PBM. Shown here are the untreated standard iADSCs, untreated IT1 control cells, experimental IT1 iADSCs exposed to NIR-G PBM consecutively, untreated IT2 control cells, experimental IT2 iADSCs exposed to NIR-G PBM consecutively, untreated IT3 control cells, and experimental IT3 iADSCs exposed to NIR-G PBM consecutively. Scale bar: 500 *μ*m.

**Figure 7:**
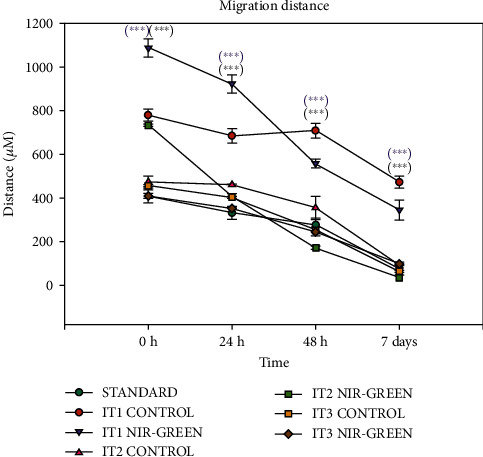
Migration analysis of transdifferentiated iADSCs 0 h, 24 h, 48 h, and 7 days post-PBM. The IT1 NIR-G PBM iADSCs significantly (*P* < 0.001) migrated over time compared to the standard and control.

Migration was measured with the “central scratch method”. The migration rate of each iADSC group was studied 0 h, 24 h, 48 h, and 7 days post-PBM as the “wound” closed. Groups showed a linear growth pattern in homing progression. Cells treated with NIR-G PBM resulted in significant augmentation of cellular migration (Figure 6). Statistical analysis of cell migration showed that the control and NIR-G PBM-treated iADSCs had a significant (*P* < 0.001) decrease in distance migrated post-PBM compared to the standard (Figure 7). Cell treated with IT1 NIR-G PBM showed the most significant (*P* < 0.001) decrease in scratch width of these groups. The results also indicated that neither transdifferentiation media nor PBM negatively impacted migration abilities in treated iADSCs.

### 3.3. Biochemical Analysis

#### 3.3.1. Viability: MTT Assay

**Figure 8:**
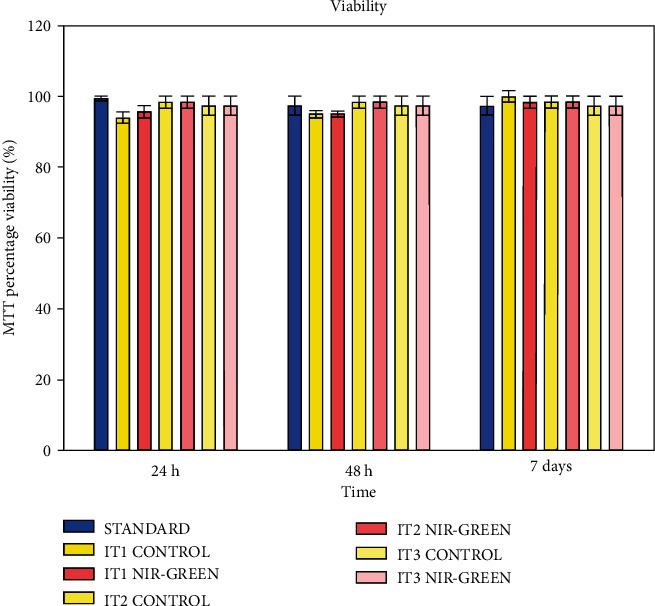
Cell viability studies of transdifferentiated iADSCs 24 h, 48, and 7 days post-PBM. MTT assay percentage viability. All cells maintained high viability, and no significant increases or decreases were recorded.

The MTT assay was used to determine the percentage cell viability after transdifferentiation and PBM. Results indicated no significant effects on the viability of the cells compared to the standard or control groups (Figure 8). High viability was maintained over time.

#### 3.3.2. DNA Synthesis

**Figure 9:**
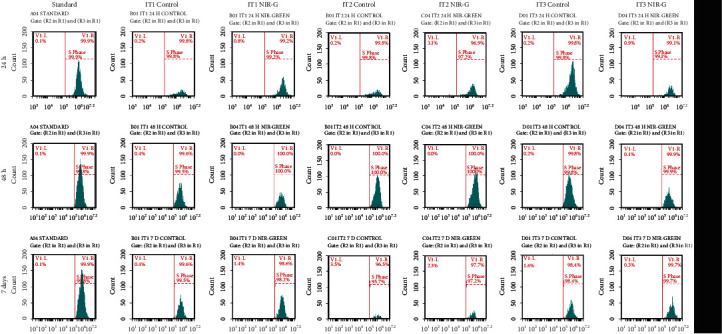
EdU results of IT3 transdifferentiating iADSCs 24 h, 48 h, and 7 days post-PBM. S phase distribution using EdU base-click analysis. A shift to the right in distribution indicated an increase in EdU expression.

The S phase was determined by EdU flow cytometry to measure the percentage of DNA synthesis [20]. Immortalized ADSCs may terminally transdifferentiate instead of reentering the cell cycle for proliferation at the G1 phase [21, 22]. Immortalized ADSCs maintained high percentages (>98%) of DNA synthesis over time and irrespective of the treatment (Figure 9).

### 3.4. Protein Detection

#### 3.4.1. ELISA

**Figure 10:**
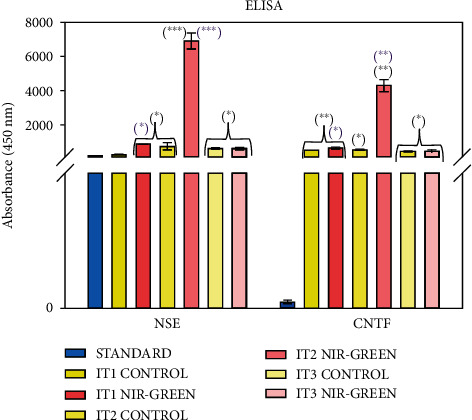
Protein detection ELISA results of IT3 transdifferentiated iADSCs using NSE and CNTF 7 days following PBM. All treated iADSCs showed an increase in NSE and CNTF expression compared to the standard and control groups.

Immortalized ADSC protein detection of early, NSE, and late, CNTF, neuronal markers was determined by ELISA 7 days post-PBM (Figure 10). Results indicated that IT1 NIR-G PBM-treated iADSCs had a significant increase in NSE (*P* < 0.001) and CNTF (*P* < 0.05). Induction type 2 PBM-treated cells had a significant increase in NSE (*P* < 0.01) and CNTF (*P* < 01) expression compared to the standard and control. Finally, iADSCs exposed to IT3 and consecutive PBM indicated a significant upregulation in NSE (*P* < 0.001) compared to the standard and a significant increase in CNTF compared to the standard (*P* < 0.001) and control (*P* < 0.01). Cells treated with IT2 and consecutive PBM showed the most NSE and CNTF expression increase compared to all the experimental groups; thus, this group was used for subsequent RT-PCR studies.

### 3.5. Genetic Expression

#### 3.5.1. RT-PCR

**Figure 11:**
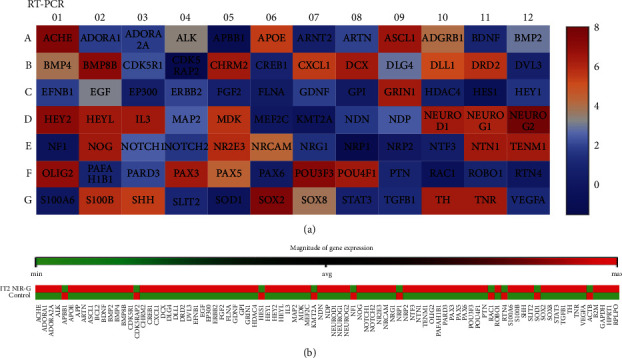
Real-time PCR of NIR-G transdifferentiated iADSCs. (a) Illustrative heat map indicated the fluorescent intensity detection of each gene generated with GeneGlobe software, where red corresponds to high detection levels in the experimental group. (b) Clustergram compared the IT2 NIR-G treated group averages to the control averages, where red indicates high expression levels and green indicates low levels of expression.

This RT-PCR experiment was performed on IT2 NIR-G PBM-treated cells and subsequently compared to a control group treated with IT2 but not PBM. The RT-PCR array detected 84 genes associated with neurogenesis and transdifferentiation including the cell cycle, proliferation, migration, adhesive properties, and synapsis.  Figure 11 is a heat map that indicates the RT-PCR results; the heat map related each gene to its degree of expression where blue showed low levels of gene expression and red showed high levels of gene expression. This RT-PCR array detected genes related to transdifferentiating cells which was indicative of successful transdifferentiation of IT2 NIR-G PBM of iADSCs into neuronal-like cells. Genes related to homing, including DCX and MDK, were also expressed, agreeing with the above migration study. A gene related to iADSC remodelling, BMP8B, for neuronal purposes was also expressed.

## 4. Discussion

Following analysis, results revealed that IT2 NIR-G (5.1%), IT1 NIR-G and IT2 control (13.9% and 13.0%, respectively), and IT2 NIR-G (5.4%) had the most significant (*P* < 0.05) decline in CD44, CD90, and CD166 expression compared to the standard and all experimental groups. At time of writing, no studies mention the effect of neuronal differentiation on CD [7, 23, 24]. Ashjian and coworkers, who formulated IT1, and Cardozo and coworkers characterized ADSCs prior to differentiation but did not perform CD marker characterization after differentiation [23, 24]. A previous study using IT2 without PBM screened for CD90 prior to implementation of differentiation but did not comment on its detection following differentiation. Previous studies that also used RA, forskolin, and indomethacin for differentiation detected CD44, CD90, and CD166 prior to differentiation but were not tested for following induction [23, 25, 26]. Moraes and coworkers demonstrated the downregulation of CD44, CD90, and CD166 when mesenchymal SCs were differentiated into adipogenic and osteogenic lineages; this has not been performed in neuronal differentiation studies [27].

Ashjian and coworkers who formulated IT1 screened for NeuN, NSE, and MAP2 [23]. They detected an increase in NSE expression and observed that NeuN did not significantly increase compared to the control, and no late neuronal markers were detected. Cardozo and coworkers detected an upregulation in the expression of early neuronal markers TUB-III, GFAP, Nestin, and synaptophysin [24]. Cardozo and coworkers did not screen for NSE nor any late neuronal markers [24]. A study by Jang and coworkers transdifferentiated ASCs into neural-like cells through forskolin and observed an upregulation in NSE and MAP2 expression [26]. Immortalized ADSCs were transdifferentiated into neuronal-like cells with RA and resulted in NeuN and MAP2 expression [25]. Immunoreactivity with NeuN and NSE was significantly (*P* < 0.05) higher in iADSCs treated with IT2 NIR-G PBM (85.7% and 78.0%) compared to the other experimental groups. The detection of early neuronal markers is not unusual in neuronal transdifferentiation studies; however, the experimental groups showed an increase in early markers compared to each respective control group due to PBM stimulation [23, 24]. Additionally, the late neuronal markers, MAP2 and Tau, were significantly (*P* < 0.05) expressed with IT2 NIR-G (79.0% and 84.0%). The expression of MAP2 and Tau as observed in this study differs from previous studies that induced transdifferentiation without PBM and did not detect any late neuronal markers [23, 24]. This study demonstrated that PBM, combined with differentiation media, specifically, IT2 NIR-G-treated iADSCs, augmented neurogenesis and resulted in more mature neuronal-like cells compared to the standard and control groups. Flow cytometry confirmed that the IT2 NIR-G group expressed the highest percentage of early and late neuronal markers with the largest decrease in stemness.

It was observed that IT2 showed the largest change in morphology compared to the standard, IT1 and IT3. Furthermore, IT2 treated with consecutive NIR-G PBM showed the most distinct morphological changes. As described by Cardozo and coworkers, the shrinking towards the nucleus and extending branches was observed [28]. Additionally, the bulbous formation of the cellular body with axon hillock similar formations as described by Singh and coworkers after transdifferentiating MSCs into neurons was also observed [29].

The homing ability of cells is a crucial element in transplanting transdifferentiated iADSCs to the site of injury [30]. The cell homing rate was measured with the “central scratch assay”, and the distance that cells migrated to “heal” the artificial wound was determined. Overall, laser light stimulated the migration of the differentiated cells compared to the standard. When the different experimental groups were compared, cells treated with IT1 and NIR-G PBM indicated a significant (*P* < 0.001) decrease in wound closure compared to all other groups. Nöth and coworkers observed that cells migrated when exposed to IT1 [31]. Several studies have observed that the addition of RA, EGF, and/or bFGF to transdifferentiation media significantly upregulates cell migration [32–35]. Multiple studies have shown that cellular migration is augmented when applying PBM at 804 nm [36], 516 nm, and 635 nm [37]. It has been observed that SCs preconditioned with PBM displayed higher rates of migration once transplanted [38].

Transdifferentiated iADSC viability was determined with the MTT assay. Results indicated that cell viability was maintained irrespective of treatment; therefore, it was concluded that neither transdifferentiation nor PBM had detrimental effects on cell health. The outcome of IT1 differs to that of Ning and coworkers who observed apoptosis 24 h following IT [39]. However, cells exposed to transdifferentiation media containing EGF, bFGF, RA, or forskolin showed maintenance in cell viability [33, 35, 40, 41]. It has been established that PBM does not have a significant stimulatory or inhibitory effect on iADSC viability [18, 42]. Studies using visible laser light at 470 nm and 410 nm reported no detrimental effects on cellular viability [43, 44]. Near-infrared laser light at 890 nm showed no effects on bone-marrow MSCs post-PBM [45]. Finally, iADSCs exposed to consecutive PBM at 825 nm and 525 nm have also showed no effects on cell viability [18].

EdU detection by flow cytometry was used to determine DNA synthesis in the S phase. As EdU (5-ethynyl-2′-deoxyuridine) is analogous to thymidine and incorporates into DNA during active DNA replication, it can be used to detect the percentage of cells in the S phase [20, 46], and it is also a more robust alternative to BrdU [46]. In this study, transdifferentiating cells were maintained in the S phase over time regardless of treatment; this is like the transdifferentiation EdU results of Chehrehasa and coworkers [47]. Previous studies have shown that cells exposed to transdifferentiation media containing indomethacin, insulin, and/or IBMX increased BrdU expression indicating DNA synthesis [48–50]. A decrease in the S phase was observed by Encinas and coworkers when SH-SY5Y neuroblastoma cells, treated with RA (10 *μ*M) and BDNF (50 ng/ml), transdifferentiated into homogenous neuronal-like cells [51]. Additionally, the authors of a previous study transdifferentiated rat NPCs with (10 ng/ml) and EGF (10 ng/ml) and observed high expression of BrdU; this led to the conclusion that the NPCs were immature and in the S phase to synthesize DNA [52]. Furthermore, studies that transdifferentiated various cell types with bFGF, EGF, *β*ME, RA, and/or BHA indicated a maintenance of high BrdU expression [51, 53, 54]. In this study, a decrease in S phase distribution was not observed; therefore, it was concluded that the iADSCs had not terminally transdifferentiated and were actively synthesizing DNA for proliferation. Terminal transdifferentiation can be defined as the phase in the cell cycle in which proliferation stops for cellular specialization; thus, DNA synthesis decreases as cells commit towards terminal transdifferentiation [55].

Early, NSE, and late, CNTF, neuronal marker expression was detected by ELISA. The increase in NSE expression in IT1-, IT2-, and IT3-treated iADSCs was consistent with previous studies [23–26]. A study by Ashjian and coworkers observed a significant increase in NSE after exposing isolated ADSCs to IT1 for 14 days without PBM treatment; however, they did not detect any late neuronal markers [23]. A study that incubated ADSCs for 14 days in IT2 media without PBM also observed NSE expression, but not CNTF [24]. Forskolin was used to transdifferentiate ASCs to neural-like cells and observed an upregulation in NSE, early, and MAP2, late neuronal markers [26]. Retinoic acid upregulated NeuN and MAP2 expression when ADSCs transdifferentiated into neuronal-like cells [25]. Multiple studies have observed an increase in late neuronal marker expression including MAP2, TUB-III, GFAP, or Nestin when ADSCs were transdifferentiated with RA, forskolin, or indomethacin [23, 25, 26]. Transdifferentiating studies performed without the use of PBM often showed little to no detection of late neuronal markers [7, 23, 24, 56]. This study established the remarkable effects of laser light when applied for iADSC transdifferentiation purposes to neuronal-like cells.  Table 2 is a summary of the functions of highly expressed genes with the corresponding *P* value and fold change compared to the control from the RT-PCR results. It was concluded that NIR-G PBM vastly increases the transdifferentiation capabilities of IT2-treated iADSCs.

## 5. Conclusion

Results from this study showed that PBM, specifically NIR-G PBM, increased the transdifferentiation abilities of iADSCs when combined with differentiation media. Morphology observations and characterization also showed the multipotent plasticity capabilities of iADSCs for neuronal-like transdifferentiation. Furthermore, immunofluorescent laser light augmented early and late neuronal marker expression while simultaneously decreasing the expression of SC markers. Biochemical assays showed that iADSCs maintained cell viability irrespective of treatment. Genetic expression of NSE and CNTF was detected through ELISA and showed augmentation in all experimental groups but was most augmented in the IT2 NIR-G PBM-treated group. The IT2 NIR-G group was overall the most augmented and following RT-PCR analysis showed an increase in genes pertaining to neurogenesis, transdifferentiation, and neuronal-like plasticity. In conclusion, optimization of the transdifferentiation process of iADSCs into neuronal-like cells was observed when consecutive 825 nm + 525 nm PBM was applied. Future directives to conclude on terminal neuronal differentiation and function should further be confirmed using electrophysiology and sodium ion channel protein expression. By optimizing the transdifferentiation process of ADMSCs, it can benefit future *in vivo* studies and clinical studies with regard to neurodegenerative disorders. Successful implementation of this protocol could potentially lead to the production of transplants to aid in the repair of mechanical brain injuries and neurodegenerative disorders. The use of consecutive PBM applied to transdifferentiation and maintenance iADSCs has great potential for the field of regenerative medicine.

## Figures and Tables

**Table 1:** Laser parameters.

	Near infrared (NIR)	Green (G)
Wavelength (nm)	825	525
Type	Diode	Diode
Emission	CW	CW
Power (mW)	570	574
Power density (mW/cm^2^)	59.24	59.66
Fluence (J/cm^2^)	5	5
Time of irradiation (s)	1 min 25 s	1 min 23 s
Spot size (cm^2^)	9.62	9.62

**Table 2:** Genes and transcription factors related to neurogenesis detected through RT-PCR following IT2 NIR-G PBM treatment with their relevant functions, fold change, and *P* values.

Gene	Function	Fold change	*P* value	References
ACHE	Assists in neurotransmission	170.62	0.300763	[57]
APOE	Provides neuronal protection and repair after injury	43.96	0.037188	[58]
ASCL1	Neuronal commitment in differentiation	99.59	0.015643	[59]
BMP4	Stimulates neuronal differentiation via ERK pathway	7.75	0.004901	[60]
BMP8B	Mediates remodelling of the neurovascular network in ADSCs	139.55	0.245330	[61]
CHRM2	Excitation of neurons, synaptic plasticity, and feedback regulation of ACHE	87.91	0.036105	[62]
CXCL1	Critical role in migration in the brain; allows for BBB permeabilization	45.45	0.035850	[7]
DCX	Assists in neuronal homing	87.91	0.036105	[63]
DLL1	Regulates Notch signalling; mediates cell fate determination	36.79	0.039993	[64]
DRD2	Synthesis and regulation of dopamine; synaptic plasticity	53.13	0.037051	[65]
GRIN1	Regulation of synapsis	87.91	0.036105	[66]
HEY2	Key role in brain development	138.59	0.182292	[67]
HEYL	Promotes neuronal differentiation of NPCs	87.91	0.036105	[68]
IL3	Regulates proliferation and survival of NPCs	87.91	0.036105	[69]
MDK	Assists in proliferation and migration	51.20	0.036887	[70]
NEUROD1	Essential for the survival and differentiation of newborn neurons	87.91	0.036105	[71]
NEUROG1	Encourages neurite outgrowth	71.08	0.095615	[72]
NEUROG2	Regulates differentiation of NPCs	264.66	0.353172	[73]
NOG	Required for proper CNS development	87.91	0.036105	[74]
NR2E3	Maintains and regulates neural stem and neocortex development	58.40	0.037037	[75]
NRCAM	Responsible for neuronal cell adhesion	20.08	0.015638	[76]
NTN1	Plays a role in axon guidance and cell homing during neuronal network development	87.91	0.036105	[77]
TENM1	Organizes the synaptic network by matching synaptic partners: axons and target projection neuronal cells	87.91	0.036105	[78]
OLIG2	Determines motor neuron and oligodendrocyte differentiation; sustains replication in early neuronal development; regulates proliferation of NPCs	118.99	0.005325	[79]
PAX3	Impacts proliferation, survival, differentiation, and motility	87.91	0.036105	[80]
PAX5	Provides neuroprotection and aids in the healing of injured neurons	21.73	0.050238	[81]
POU3F3	Involved in CNS development	147.51	0.123322	[82]
POU4F1	Important for differentiation, axonal elongation, and cell survival	87.91	0.036105	[83]
S100B	Aids in normal CNS formation and recovery following injury	56.68	0.037128	[84]
SHH	Promotes proliferation and differentiation	44.16	0.037775	[85]
SOX2	Essential in self-renewal maintenance of NSCs; directs the differentiation of SCs to neuronal differentiation	180.77	0.218528	[86]
SOX8	Aids in NPC specification and cell survival	14.37	0.020562	[87]
SOD1	Protects the cell from ROS	0.85	0.614984	[88]
TNR	Encourage neurite outgrowth and neural cell adhesion, proliferation and migration, axonal guidance, myelination, and synaptic plasticity	87.91	0.036105	[89]

## Data Availability

The raw and analysed data used to support the findings of this study are available from the corresponding author upon request.
